# Prognostic value of neuron-specific enolase in patients with advanced and metastatic non-neuroendocrine non-small cell lung cancer

**DOI:** 10.1042/BSR20210866

**Published:** 2021-08-02

**Authors:** Peng Yan, Yiming Han, Anna Tong, Jie Liu, Xinyu Wang, Chuanyong Liu

**Affiliations:** 1 Department of Oncology, Jinan Central Hospital, Cheeloo College of Medicine, Shandong University, Jinan, P.R. China; 2 Graduate School, Shandong First Medical University and Academy of Medical Sciences, Jinan, P.R. China; 3 Department of Radiation, The 960th Hospital of the PLA Jonit Logistics Support Force, Jinan, P.R. China

**Keywords:** biomarkers, Heterogeneity, lung cancer, Prognosis

## Abstract

**Background:** Increased serum neuron-specific enolase (NSE) level was found in a substantial proportion (30–69%) of patients with non-small-cell lung cancer (NSCLC), but little was known about the clinical properties of NSE in NSCLC.

**Objective:** We aimed to assess the level of serum NSE to predict prognosis and treatment response in patients with advanced or metastatic non-neuroendocrine NSCLC.

**Methods:** We retrospectively analyzed 363 patients with advanced and metastatic NSCLC between January 2011 and October 2016. The serum NSE level was measured before initiation of treatment.

**Results:** Patients with high NSE level (≥26.1 ng/ml) showed significantly shorter progression-free survival (PFS) (5.69 vs 8.09 months; *P*=0.02) and significantly shorter overall survival (OS) than patients with low NSE level (11.41 vs 24.31 months; *P*=0.01).

NSE level was an independent prognostic factor for short PFS (univariate analysis, hazard ratio [HR] = 2.40 (1.71–3.38), *P*<0.001; multivariate analysis, [HR] = 1.81 (1.28–2.56), *P*=0.001) and OS (univariate analysis, [HR] = 2.40 (1.71–3.37), *P*<0.001; multivariate analysis, [HR] = 1.76 (1.24–2.50), *P*=0.002).

**Conclusion:** The survival of NSCLC patients with high serum NSE level was shorter than that of NSCLC patients with low serum NSE levels. Serum NSE level was a predictor of treatment response and an independent prognostic factor.

## Background

Worldwide, lung cancer is the leading cancer in terms of incidence and mortality, with 2.2 million new lung cancer cases and 1.8 million deaths in 2020 [1]. Traditionally, lung cancer has been divided into two main histological types: small cell lung cancer (SCLC) accounts for 15–25% of all lung cancer cases, and non-small-cell lung cancer (NSCLC) accounts for the remaining 75–85% [2], the latter is mainly subdivided into adenocarcinoma, squamous cell carcinoma (SCC), and other subtypes, including sarcomatoid carcinoma and neuroendocrine large cell carcinoma, are uncommon variants [3]. Despite advances in early detection and standard therapies, approximately 57% of patients are diagnosed at an advanced stage and have a poor prognosis, with an overall 5-year survival rate of 10–15% [4].

Serum biomarkers provide valuable information about the diagnosis and prognosis of a wide variety of malignant tumors, and the best known and most widely studied tumor markers of lung cancer are carcinoembryonic antigen (CEA), SCC antigen, cytokeratin-19 fragments (Cyfra21-1) and neuron-specific enolase (NSE) [5,6]. Serum NSE has been established as a clinically useful marker for staging, monitoring treatment and predicting relapse of SCLC [7].

Increased serum NSE levels were found in a substantial proportion (30–69%) of patients with NSCLC [8,9]. However, few studies had assessed the clinical value of NSE elevation in NSCLC, especially SCC and adenocarcinoma. Therefore, the purpose of the present study was initially to explore the role and value of NSE in predicting prognosis in advanced and metastatic non-neuroendocrine NSCLC patients (SCC and adenocarcinoma).

## Methods

### Patients

We reviewed NSCLC patients who underwent treatments at our institution between January 2011 and October 2016. We drafted the inclusion and exclusion criteria.

Eligible patients were enrolled according to the following criteria: (1) patients with a histological diagnosis of SCC or adenocarcinoma; (2) patients with complete clinical and histological information as well as follow-up data; (3) patients aged > 18 years; (4) patients with no previous treatment for cancer; (5) patients with complete tumor marker data; (6) patients with clinical stage IIIB, IIIC, IVA and IVB disease defined by the tumor node metastasis staging guidelines (TNM, Eighth Edition of the American Joint Committee on Cancer, AJCC-8th).

The exclusion criteria were as follows: (1) the patient had other malignancies; (2) the patient had systemic infection, autoimmune disease or inflammation; (3) the patient had an Eastern Cooperative Oncology Group (ECOG) score ≥ 2; (4) the patient had any positive immunohistochemical staining for CgA, Syn or CD56.

Finally, 363 patients remained and were analyzed in the present study. The study was approved by the Ethical Committee of Jinan Central Hospital Affiliated to Shandong University. Due to the retrospective nature of the study, informed consent was obtained verbally from the subjects’ family members or guardians. The ethics committee approved this procedure. The present study was conducted in accordance with the Declaration of Helsinki.

### Treatment and follow-up

Eighty-seven patients with EGFR mutations used gefitinib 250 mg/day orally or erlotinib 150 mg/day orally as first-line therapy, and the other patients used platinum-based doublet therapy. Patients were treated with chemotherapy, which was composed of four to six cycles. Bevacizumab was combined or not according to the principle of individualization. Comprehensive evaluation was performed after every two cycles of chemotherapy. After chemotherapy cycles were completed, the follow-up periods were set as every 3–4 months for the first and second years, every 6 months for the third year and annually thereafter.

The post-treatment surveillance program consisted of physical examination, chest computed tomography (CT), brain magnetic resonance imaging (MRI), bone emission CT scans and additional tests as needed to confirm patient response and to assess disease progression. All responses were defined according to the revised Response Evaluation Criteria in Solid Tumors version 1.1 (RECIST 1.1) [10]. The objective response rate (ORR) referred to the percentage of patients who had complete response (CR) or partial response (PR). The disease control rate (DCR) referred to the percentage of patients who had CR, PR or stable disease (SD). Progression-free survival (PFS) was measured from the date of initiation of treatment to either the date of disease progression or death. Overall survival (OS) was defined as the interval from the date of initiation of treatment to the date of death from any cause. PFS was the primary ending. Data collection was terminated in October 2019.

### Data collection

The main clinical characteristics, such as age, gender, smoking history and pathological differentiation, clinical stage, ECOG score and NSE level were extracted from medical records. AJCC-8^th^ TNM classification was used to define TNM stage.

Serum NSE was detected by electrochemiluminescence immunoassay on a Roche Analytics E170 Immunology Analyzer (Roche Diagnostics, China). The primary steps were as follows. Blood samples for serum NSE assay were centrifuged for 10 min at 3500 rpm. The Elecsys NSE assay for Modular analyzer used an mAb-labeled with biotin and another mAb coupled with Ruthenium. In the presence of the antigen (NSE), immunocomplexes were immobilized on to the surface of the electrode with magnetic beads labeled with streptavidin. Application of an electric voltage to the electrode then induced chemiluminescence detected by a spectrophotometer. The assay was performed following the directions given by the manufacturer. Results were expressed in nanograms per milliliter (ng/ml).

Because SCC had a low EGFR mutation rate, only adenocarcinoma required EGFR detection.

### Expression analysis of NSE in NSCLC database

RNA sequencing data of The Cancer Genome Atlas (TCGA) NSCLC cohort were downloaded from TCGA data portal (https://tcga-data.nci.nih.gov/tcga/). The NSE messenger RNA (mRNA) expressions in NSCLC tissues and normal tissues were analyzed. Gene set enrichment analysis (GSEA) was performed using GSEA software v2.0.13 (http://software.broadinstitute.org/gsea/).

### Statistical analysis

The X-tile program was used to calculate the optimal cut-off value for NSE. The Kolmogorov–Smirnov test was used to reveal a normal distribution. The Chi-squared test was used to compare the clinical characteristics grouped by NSE. OS and PFS were calculated and depicted using the Kaplan–Meier method and compared using the log-rank test. The Kaplan–Meier method was used to calculate brain metastasis. A Cox proportional hazards model was applied to explore the risk factors for PFS and OS, with proportional hazard ratio (HR) and 95% confidence interval (CI). *P*<0.05 was regarded as statistically significant. All statistical analyses were conducted with SPSS Statistics 25 (IBM Corporation, NY, U.S.A.).

## Results

### Baseline characteristics

From January 2011 to October 2016, 363 patients with advanced and metastatic NSCLC were enrolled based on the inclusion and exclusion criteria. The baseline characteristics are summarized in Table 1. Among these 363 patients, 239 (65.84%) patients were male and 124 (34.16%) were female, and age ranged from 27 to 95 (mean 66.1 ± 11.4). Based on the TNM staging system, 75 patients presented with stage IIIB, followed by 9 with IIIC, 146 with stage IVA, and 133 with stage IVB. At the time of diagnosis, 53 patients (14.60%) had brain metastases. Regarding the pathological differentiation level of tumors, 22.59, 48.21 and 29.20% of patients were histologically diagnosed with well, moderately and poorly differentiated disease, respectively. Eighty-seven patients were with EGFR mutations, and 104 adenocarcinomas were EGFR-wildtype or unknown. The average value of NSE level was 20.1 ng/ml (range 0.1–285.2 ng/ml).

**Table 1:** Baseline characteristics of the enrolled patients

Characteristics	Number of patients
Overall (%)	363 (100)
Age (years)	
<67	176 (48.49)
≥67	187 (51.51)
Mean ± S.D	66.1 ± 11.4
Range	27–95
Gender, *n* (%)	
Male	239 (65.84)
Female	124 (34.16)
Smoking history, *n* (%)	
Former/current	219 (60.33)
Never	144 (39.67)
ECOG score	
0	224 (61.71)
1	139 (38.29)
Histological types, *n* (%)	
Adenocarcinoma	191 (52.62)
SCC	172 (47.38)
Clinical stage, *n* (%)	
IIIB	75 (20.66)
IIIC	9 (2.48)
IVA	146 (40.22)
IVB	133 (36.64)
Pathological differentiation, *n* (%)	
Well	82 (22.59)
Moderate	175 (48.21)
Poor	106 (29.20)
NSE level (ng/ml)	
Mean ± SD	20.1 ± 23.7
Range	0.1–285.2
Brain metastasis	
Yes	53 (14.60)
No	310 (85.40)
EGFR status (adenocarcinoma)	
Mutation	87 (45.55)
Wildtype	80 (41.88)
Unknown	24 (12.57)

### The optimal cut-off value for NSE level

**Figure 1:**
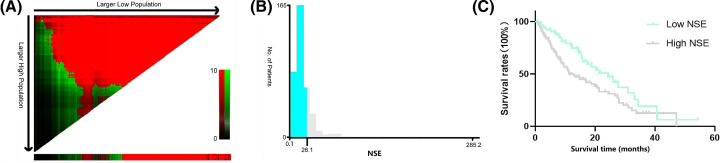
Analysis of serum NSE using X-tile Analysis of serum NSE using X-tile. The sample of patients was equally divided into training and validation sets. X-tile plots of training sets are shown in the left panels (**A**), with plots of matched validation sets shown in the smaller inset. The optimal cut-off values highlighted in left panels are shown in histograms of the entire cohort (**B**), and Kaplan–Meier plots are displayed in right panels (**C**). The optimum cut-off points for NSE level was 26.1 ng/ml according to the X-tile program.

According to the X-tile program, the optimum cut-off value for NSE level was 26.1 ng/ml (Figure 1). Based on the optimal cut-off value, patients were subsequently divided into two groups (NSE < 26.1 ng/ml and NSE ≥ 26.1 ng/ml) for further analyses.

### Correlation between NSE level and other clinicopathological characteristics

Potential correlations of NSE levels with other clinicopathological variables were explored (Table 2). The chi-squared test revealed that the NSE level was correlated with the ECOG score (*P*<0.01), histological source (*P*<0.01), pathological differentiation (*P*<0.01) and clinical stage (*P*<0.01).

**Table 2:** Correlation of serum NSE level (NSE < 26.1/NSE ≥ 26.1 ng/ml) with clinicopathological characteristics

Characteristics	Number of all patients (%)		χ²	*P*
	NSE < 26.1 ng/ml	NSE ≥ 26.1 ng/ml		
Overall	298 (100)	65 (100)		
Age (years)				
<67	147 (49.33)	29 (44.62)	0.48	0.49
≥67	151 (50.67)	36 (55.38)		
Gender				
Male	196 (65.77)	43 (66.15)	0.00	0.95
Female	102 (34.23)	22 (33.85)		
Smoking history				
Former/current	176 (59.06)	43 (66.15)	1.12	0.29
Never	122 (40.94)	22 (33.85)		
ECOG score				
0	199 (66.78)	25 (38.46)	5.42	<0.01
1	99 (33.22)	40 (61.54)		
Histological source				
Needle biopsy	162 (54.36)	41 (63.07)	11.73	<0.01
Bronchoscopy	60 (20.13)	15 (23.08)		
Resection	53 (17.79)	1 (1.54)		
Cytology	23 (7.72)	8 (12.31)		
Histological types				
SCC	91 (30.5)	14 (21.5)	2.10	0.15
Adenocarcinoma	207 (69.5)	51 (78.5)		
Clinical stage				
IIIB + IIIC	82 (27.52)	2 (3.08)	17.92	<0.01
IVA + IVB	216 (72.48)	63 (96.92)		
Pathological differentiation				
Well	70 (23.49)	12 (18.46)	42.02	<0.01
Moderate	162 (54.36)	13 (20.00)		
Poor	66(22.15)	40 (61.54)		
EGFR status (adenocarcinoma)				
Mutations	75 (48.39)	12 (33.33)	3.51	0.17
Wide	60 (38.71)	20 (55.56)		
Unknown	20 (12.90)	4 (11.11)		
Ki-67				
<0.3	68 (22.82)	9 (13.85)	2.57	0.11
≥0.3	230 (77.18)	56 (86.15)		

*P*<0.05 was considered to indicate significant difference. The *P*-value was calculated by Chi-squared test.

### Effectiveness of first-line treatment

According to RECIST 1.1 guidelines, in the low NSE group (NSE < 26.1 ng/ml), the outcomes were CR: 0.0% (0/298); PR: 18.79% (56/298); SD: 27.18% (81/298); PD: 54.03% (161/298); ORR: 18.79% (56/298); and DCR: 45.97% (137/298). In the high NSE group (NSE ≥ 26.1 ng/ml), the outcomes were CR: 0.0% (0/65); PR: 3.08% (2/65); SD: 18.46% (12/65); PD: 78.46% (51/65); ORR: 3.08% (2/65) and DCR: 21.54% (14/65) (Table 3).

**Table 3:** The responses in the two groups (NSE < 26.1/NSE ≥ 26.1 ng/ml)

Variable	Number of all patients (%)	
	NSE < 26.1 ng/ml	NSE ≥ 26.1 ng/ml
Overall	298 (100)	65 (100)
CR	0 (0.0)	0 (0.0)
PR	56 (18.79)	2 (3.08)
SD	81 (27.18)	12 (18.46)
Progressive disease	161 (54.03)	51 (78.46)
ORR	56 (18.79)	2 (3.08)
DCR	137 (45.97)	14 (21.54)

### The outcome of PFS and OS

Among 363 patients, at the end of follow-up, 199 (54.82%) patients had been confirmed dead.

The median PFS was 8.09 months (95% CI = 5.83–10.35) in the low NSE group (<26.1 ng/ml). The mPFS was 5.69 months (95% CI = 4.68–6.69) in the high NSE group (NSE ≥ 26.1 ng/ml) (*P*=0.02, log-rank test).

**Figure 2:**
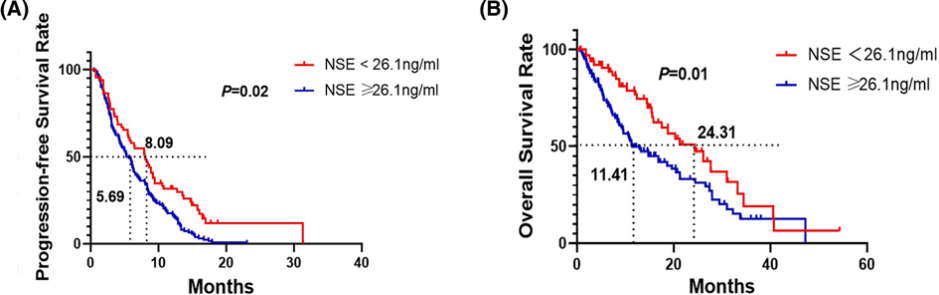
Survival was evaluated using Kaplan-Meier survival curves Kaplan–Meier survival curves for PFS (**A**) and OS (**B**) in different groups (NSE level < 26.1 ng/ml or ≥ 26.1 ng/ml) in all patients

The 1-year PFS rates of the low NSE group (NSE < 26.1 ng/ml) were 25.31%. The 1-year PFS rates of the high NSE group (NSE ≥ 26.1 ng/ml) were 10.00%, respectively (Figure 2A).

The median OS was 24.31 months (95% CI = 16.23–32.39) in the low NSE group (<26.1 ng/ml). The mOS was 11.41 months (95% CI = 7.83–14.99) in the high NSE group (NSE ≥ 26.1 ng/ml) (*P*=0.01, log-rank test).

The 1- and 2-year OS rates of the low NSE group (NSE < 26.1 ng/ml) were 76.72 and 50.87%, respectively. The 1- and 2-year OS rates of the high NSE group (NSE ≥ 26.1 ng/ml) were 49.59 and 33.16%, respectively (Figure 2B).

Of the 363 NSCLCs, 172 were SCC. The median PFS was 4.93 months (95% CI = 3.78–5.69) in the low NSE group (<26.1 ng/ml). The mPFS was 3.30 months (95% CI = 3.02–3.61) in the high NSE group (NSE ≥ 26.1 ng/ml) (*P<*0.01, log-rank test).

**Figure 3:**
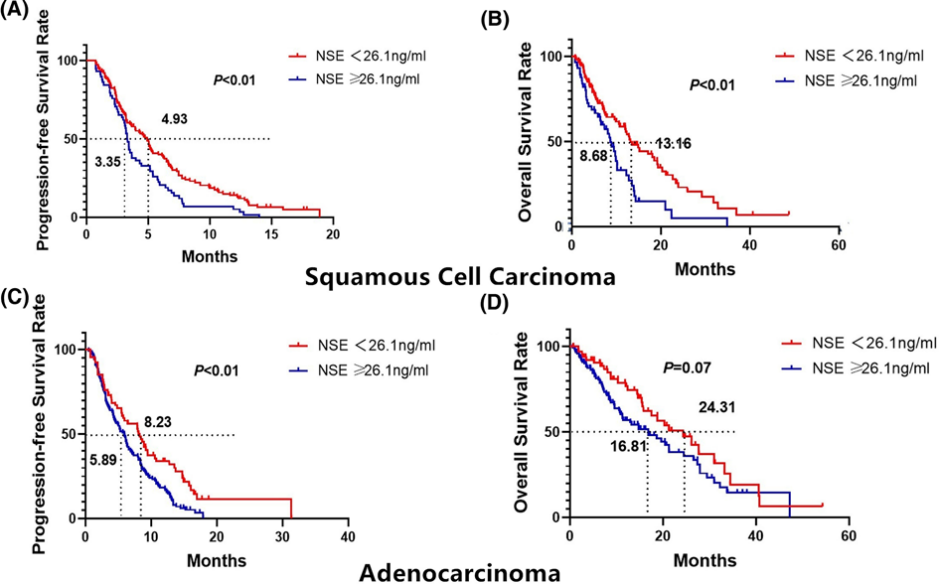
Kaplan–Meier survival curves for PFS and OS in different pathological types according to NSE level (< 26.1 or ≥ 26.1 ng/ml) (**A**) PFS probability in patients with SCC. (**B**) OS probability in patients with SCC. (**C**) PFS probability in patients with adenocarcinoma. (**D**) OS probability in patients with adenocarcinoma.

The 1-year PFS rates of the low NSE group (NSE < 26.1 ng/ml) were 13.03%. The 1-year PFS rates of the high NSE group (NSE ≥ 26.1 ng/ml) were 5.17% (Figure 3A).

The median OS was 13.16 months (95% CI = 10.20–16.12) in the low NSE group (<26.1 ng/ml). The mOS was 8.68 months (95% CI = 6.33–11.03) in the high NSE group (NSE ≥ 26.1 ng/ml) (*P*<0.01, log-rank test).

The 1- and 2-year OS rates of the low NSE group (NSE < 26.1 ng/ml) were 57.46 and 33.15%, respectively. The 1- and 2-year OS rates of the high NSE group (NSE ≥ 26.1 ng/ml) were 23.14 and 5.02%, respectively (Figure 3B).

Of the 363 NSCLCs, 191 were adenocarcinomas. The median PFS was 8.23 months (95% CI = 6.06–10.40) in the low NSE group (<26.1 ng/ml). The mPFS was 5.89 months (95% CI = 4.80–6.98) in the high NSE group (NSE ≥ 26.1 ng/ml) (*P<*0.01, log-rank test) (Figure 3C).

The 1-year PFS rates of the low NSE group (NSE < 26.1 ng/ml) were 34.00%. The 1-year PFS rates of the high NSE group (NSE ≥ 26.1 ng/ml) were 17.45%.

The median OS was 24.31 months (95% CI = 16.23–32.39) in the low NSE group (<26.1 ng/ml). The mOS was 16.81 months (95% CI = 10.73–22.89) in the high NSE group (NSE ≥ 26.1 ng/ml) (*P*=0.07, log-rank test).

The 1- and 2-year OS rates of the low NSE group (NSE < 26.1 ng/ml) were 76.72 and 56.92%, respectively. The 1- and 2-year OS rates of the high NSE group (NSE ≥ 26.1 ng/ml) were 50.87 and 38.06%, respectively (Figure 3D).

The total number of EGFR mutations in adenocarcinoma patients was 87. 104 cases were with non-mutations or unknown.

**Figure 4:**
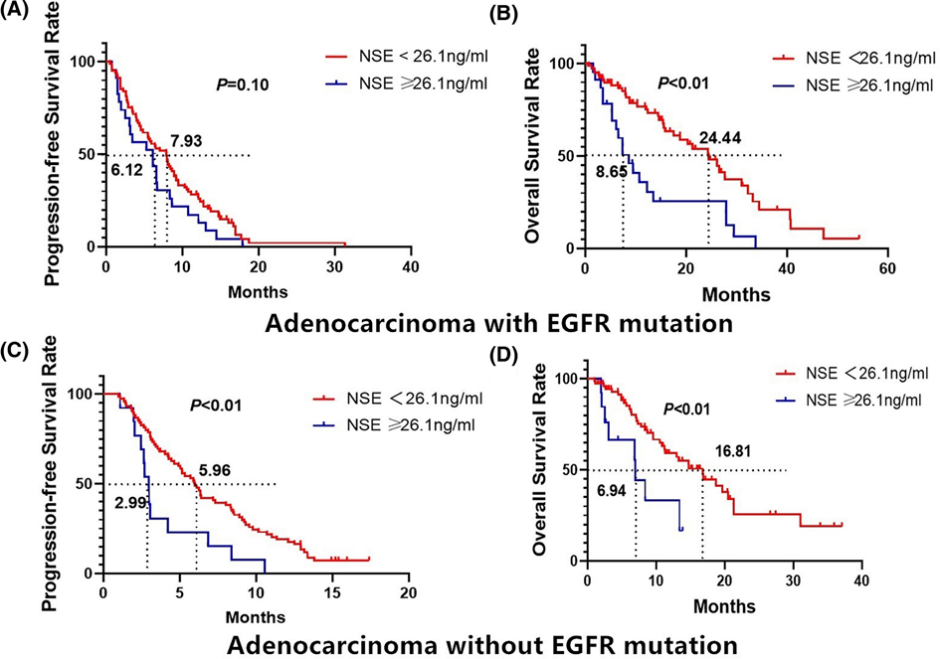
Kaplan–Meier survival curves for PFS and OS in EGFR mutation status according to NSE level (<26.1 or ≥ 26.1 ng/ml) (**A**) PFS probability in EGFR-mutations patients. (**B**) OS probability in EGFR-mutations patients. (**C**) PFS probability in EGFR-wildtype patients. (**D**) OS probability in EGFR-wildtype patients.

In EGFR mutations subgroup, patients with NSE < 26.1 ng/ml, mPFS was 7.93 months (95% CI = 5.83–10.03), and the 1-year PFS rates were 27.10%. For patients with NSE ≥ 26.1 ng/ml, the mPFS was 6.12 months (95% CI = 1.89–10.35), and the 1-year PFS were 17.39%. A significant difference in PFS was observed between the two groups (*P=*0.1, log-rank test) (Figure 4A).

The median OS of patients with EGFR mutations and low serum NSE levels (<26.1 ng/ml) was 24.44 months (95% CI = 17.33–31.55). The 1- and 2-year OS rates were 75.23 and 53.96%, respectively. The median OS of patients with EGFR mutations and high serum NSE levels (≥26.1 ng/ml) was 8.65 months (95% CI = 5.59–11.70). The 1- and 2-year OS rates were 35.81 and 25.58%, respectively. A significant difference in OS was observed between the two groups (*P*<0.01, log-rank test) (Figure 4B).

There were 80 cases of EGFR-wildtype adenocarcinomas. In this subgroup, patients with NSE < 26.1 ng/ml, mPFS was 5.96 months (95% CI = 4.88–7.10), and 1-year PFS rate was 19.04%, respectively. For patients with NSE ≥ 26.1 ng/ml, the mPFS was 2.99 months (95% CI = 2.57–3.35) and the 1-year PFS rates was 0%, respectively. A significant difference in PFS was observed between the two groups (*P*<0.01, log-rank test) (Figure 4C).

The median OS of patients with EGFR-wildtype with low serum NSE levels (<26.1 ng/ml) was 16.81 months (95% CI = 12.09–21.53). The 1- and 2-year OS rates were 59.17 and 33.32%, respectively. The median OS of patients with high serum NSE levels (≥26.1 ng/ml) was 6.94 months (95% CI = 6.66–7.22). The 1- and 2-year OS rates were 25.54 and 16.66%, respectively. A significant difference in OS was observed between the two groups (*P*<0.01, Figure 4D).

In NSCLC without brain metastasis at the time of initial diagnosis, there were 35/58 cases (60.34%) of brain metastasis in the high level NSE group and 71/252 cases (28.17%) in the low level NSE group at the end of follow-up.

In the subgroup without brain metastasis at the time of initial diagnosis, patients with serum NSE < 26.1 ng/ml, the cumulative risk of brain metastasis at 6, 12 and 18 months was 13.28, 25.34 and 34.46%, respectively.

**Figure 5:**
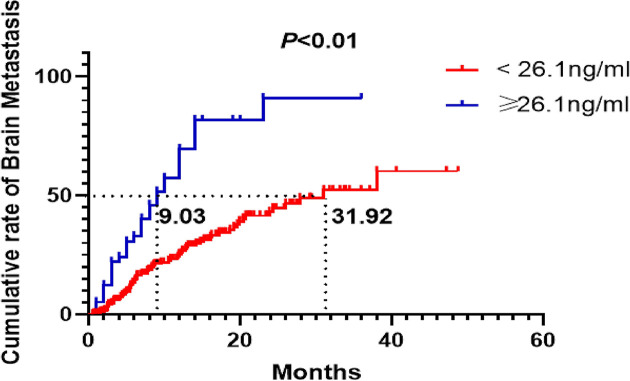
Cumulative incidence of subsequent brain metastases in patients after treatment according to NSE level (< 26.1 or ≥ 26.1 ng/ml) in patients without brain metastases at the time of initial diagnosis

In the patients with serum NSE ≥ 26.1 ng/ml, the cumulative risk of brain metastasis at 6, 12 and 18 months was 32.86, 69.55 and 81.73%, respectively. There was a significant difference between the two groups (*P*<0.01, log-rank test) (Figure 5).

### Univariate and multivariate analyses of PFS

**Figure 6:**
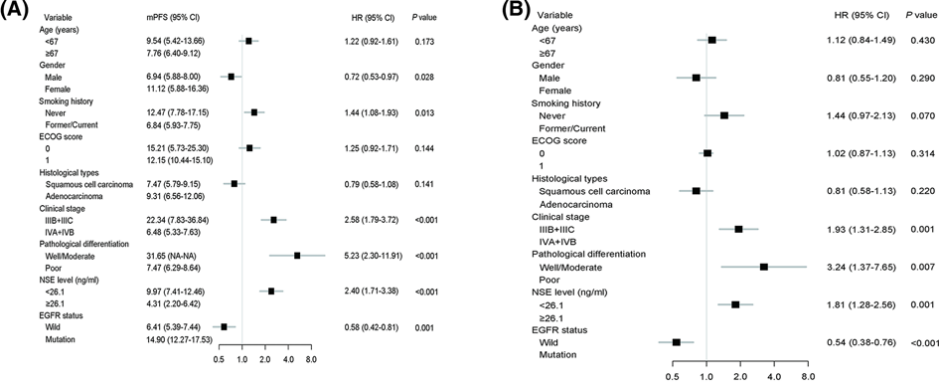
Cox proportional hazards regression analysis for PFS Result of univariate analysis (**A**) and multivariate analysis (**B**) for the association of clinical factors with PFS.

As shown in Figure 6A, the results from univariate analysis indicated that gender (HR = 0.72, *P*=0.028), smoking history (HR = 1.44, *P*=0.013), clinical stage (HR = 2.58, *P*<0.001), pathological differentiation (HR = 5.23, *P*<0.001), NSE level (HR = 2.40, *P*<0.001), and EGFR mutation status (HR = 0.58, *P*=0.001) were significant prognostic factors for PFS. Multivariate analysis indicated that clinical stage (HR = 1.93, *P*=0.001), pathological differentiation (HR = 3.24, *P*=0.007), NSE level (HR = 1.81, *P*=0.001), and EGFR mutation status (HR = 0.54, *P*<0.001) were independent prognostic parameters for PFS (Figure 6B).

### Univariate and multivariate analyses of OS

**Figure 7:**
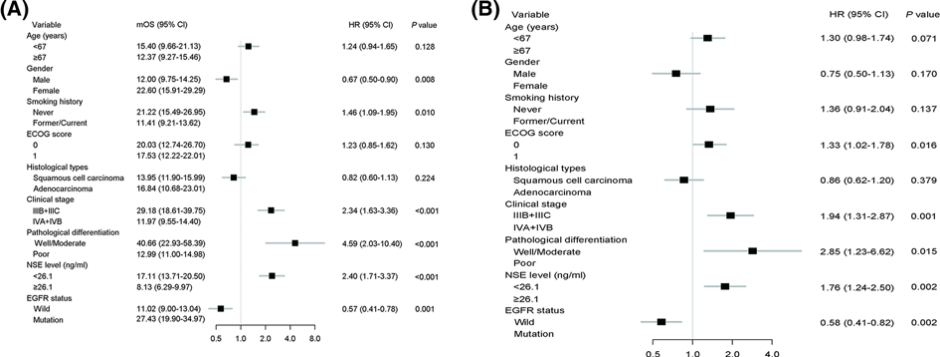
Cox proportional hazards regression analysis for OS Result of univariate analysis (**A**) and multivariate analysis (**B**) for the association of clinical factors with OS.

Univariate analysis in all patients showed that gender (HR = 0.67, *P*=0.008), smoking history (HR = 1.46, *P*=0.010), clinical stage (HR = 2.34, *P*<0.001), pathological differentiation (HR = 4.59, *P*<0.001), NSE level (HR = 2.40, *P*<0.001) and EGFR mutation status (HR = 0.57, *P*=0.001), were associated with OS (Figure 7A). To identify possible independent prognostic factors, multivariate OS analysis next was conducted in which ECOG score (HR = 1.33, *P*=0.016), clinical stage (HR = 1.94, *P*=0.001), pathological differentiation (HR = 2.85, *P*=0.015), NSE level (HR = 1.76, *P*=0.002) and EGFR mutation status (HR = 1.58, *P*=0.002) were identified as independent factors predicting OS (Figure 7B).

### GSEA of NSE in NSCLC

**Figure 8:**
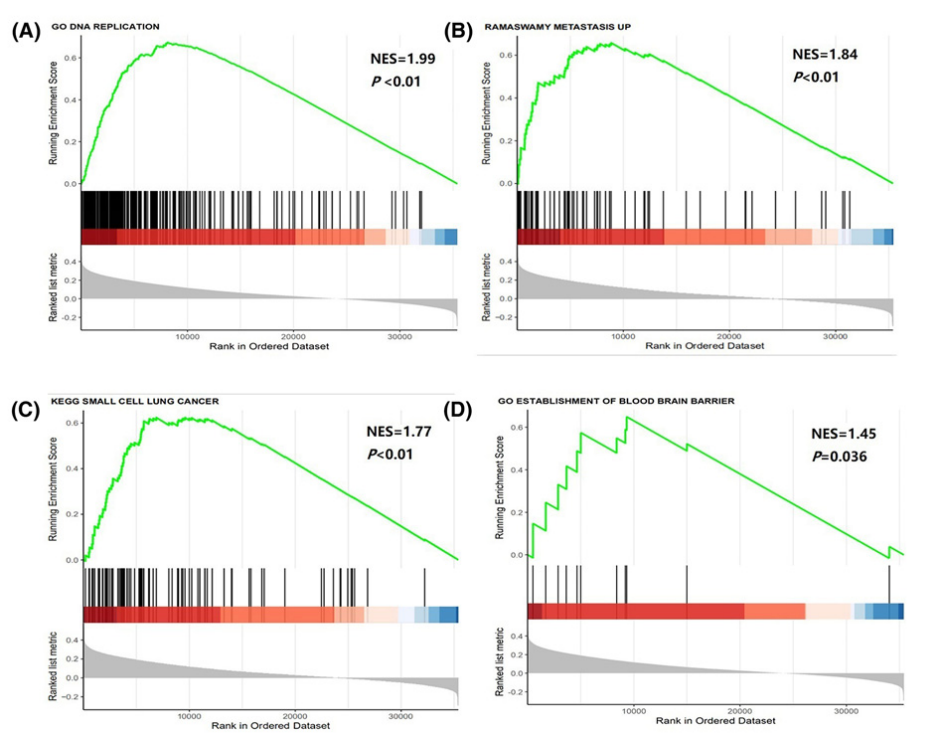
GSEA about expression of NSE transcription in NSCLC (**A**) GSEA results of DNA reproduction gene sets for high-NSE expression groups. (**B**) GSEA results of tumor metastasis gene sets for high-NSE expression groups. (**C**) GSEA results of SCLC gene sets for high-NSE expression groups. (**D**) GSEA results of passing blood–brain barrier (BBB) gene sets for high-NSE expression groups.

As shown in Figure 8, GSEA on TCGA dataset showed that NSCLC cells DNA reproduction (Normalized Enrichment Score, NES = 1.99, *P*<0.01), metastasis (NES = 1.84, *P*<0.01), signaling pathways in SCLC (NES = 1.77, *P*<0.01) and passing through the blood–brain barrier (NES = 1.45, *P*=0.036) were strongly associated with NSE expression in NSCLC cancer patients.

## Discussion

NSE, also known as enolase-γ, is neuro- and neuroendocrine-specific isoenzyme of enolase. It was originally described by Moore and McGregor in 1965 as an enzyme enriched in neurons in general and in peripheral neuroendocrine cells [11]. NSE is considered to be a useful tumor marker for tumors of neural and neuroendocrine origin, such as neuroblastoma and neuroendocrine tumors [12,13].

In the present study, we explored the significance of NSE in NSCLC. We found that the pretreatment serum NSE level is an independent prognostic and predictive factor of advanced or metastatic NSCLC patients. NSE could not only predict poor prognosis in NSCLC patients, but also indicate insensitivity to anti-tumor agents.

PFS and OS were shorter in patients with elevated NSE levels (≥26.1 ng/ml). In subgroup, both PFS and OS were shorter in patients with elevated NSE levels than those low NSE levels in SCC. In adenocarcinoma, PFS was shorter in patients with elevated NSE levels. Although there was no statistically significant difference in OS between the two groups in adenocarcinoma, a trend towards shorter survival with elevated NSE levels was observed. With EGFR mutations or not, patients with elevated pre-treatment NSE level had a shorter OS.

Some studies came to the same conclusions: a prospective study of 621 NSCLC patients showed high NSE level (>12.5 ng/ml) is a prognosticate of poor outcome [8]. Ferrigno et al. [14] enrolled 448 NSCLC patients (stage I–IV), assessed NSE before treatment and found that patients with NSE > 11 ng/ml had a worse prognosis than patients with lower NSE levels. Further, there are several studies that investigated this phenomenon in a limited number of patients. Van Zandwijk et al. [15] assessed the prognostic value of NSE and lactic dehydrogenase (LDH) in 42 locally advanced or metastatic NSCLC patients treated with chemotherapy. The present study showed that high levels of both markers (NSE and LDH) were associated with shorter survival. Another study with 84 NSCLC patients found that a high serum NSE concentration conferred lower survival and greater probability of relapse after radical surgery than a low serum NSE concentration [16].

The cut-off value of NSE in the above studies was lower than that defined by us, which may be related to the inclusion of patients included earlier stage patients in the above studies. In our study, we just focused on locally advanced or metastatic NSCLC.

We speculated the reasons of high-serum NSE predicting poor prognosis might be:

1. NSE is considered a key enzyme in glycolysis, and it plays an important role in aerobic glycolysis [17]. Cells with high NSE expression proliferated more quickly.
2. It is possible that NSCLC with high NSE is mixed with small cell components. A mixed SCLC-NSCLC component is present in a certain proportion of lung cancers [18]. The prognosis of SCLC is worse than that of NSCLC, and the 5-year relative survival for SCLC (6%) is lower than that for NSCLC (23%) for all stages [19].
3. Patients with elevated NSE levels have later stages. In the present study, Chi-squared test showed a correlation between NSE and staging.
4. In our study, high NSE levels are more frequently associated with brain metastases. The possible reason for this phenomenon is that NSCLC with neuroendocrine differentiation are more aggressive [20,21]. A study also observed the same situation—a high level of serum NSE might be associated with brain metastases in patients with lung cancers [22].
5. Among patients with EGFR mutations, those with high NSE have a shorter time to disease control time. Similar conclusions have been found in other studies. Suh et al. [23] determined that patients with elevated NSE levels (>16.3 ng/ml) had a median PFS of 10.5 months, which was shorter than that for patients with low NSE levels (<16.3 ng/ml) who had a median PFS of 15.4 months after EGFR-tyrosine kinase inhibitors (TKIs) treatment. Fiala et al. [24] reported that in 163 patients with advanced NSCLC treated with EGFR TKIs, high pretreatment serum NSE levels (>12.5 ng/ml) were associated with short PFS.
6. Histologic transformation from NSCLC to SCLC occurs, after EGFR-TKIs therapy, which is a known mechanism of resistance to first-generation EGFR-TKIs that dramatically impacts patient prognosis [25,26]. We hypothesized that patients with elevated levels of NSE may be more prone to this transformation.
7. Data from TCGA database confirmed that NSCLC cells with high NSE expression were more aggressive. GSEA revealed that NSCLC cells with high NSE expression proliferated more rapidly, are more prone to metastases, particularly brain metastases. The signal pathways are more similar to those of SCLC. This confirmed our conclusion at the molecular level.

There are some limitations in our study. First, for more than 80% patients whose tissues were obtained by percutaneous transthoracic biopsy, exfoliative cytological examination or bronchoscopy, histologic heterogeneity was hard to assess because of the limited amount of tissue. Biopsy of both primary lesions and metastatic lesions should be performed to avoid missing lung neuroendocrine components. Second, NSE level was assessed only during the pretreatment period, and was not followed at the time of disease progression. Third, re-biopsy was not performed at the time of disease progression to detect transformation to small cell carcinoma or neuroendocrine phenotype. In addition, the retrospective design of our study may have led to selection bias.

The present study had two clinical implications: one is that NSCLC patients with high NSE levels have a poor prognosis, and the other is that multisite biopsy should be performed for NSCLC with elevated NSE levels, which is possible to facilitate a precise pathological diagnosis.

### Prospection

NSCLC patients with elevated NSE are less sensitive to treatment, and it is worth exploring whether more intensive treatments, such as concurrent radiotherapy and chemotherapy are needed. In addition, as we known, patients with high mutation loads are more susceptible to immune checkpoint inhibitors than the patients with low mutation loads in lung cancers [27]. We are also interested in exploring the efficacy of NSE-elevated NSCLC treated by immune checkpoint inhibitors.

## Data Availability

Some data generated or used during the study are available from the corresponding author by request. The other data generated or used during the study are available online in TCGA data portal (https://tcga-data.nci.nih.gov/tcga/).

## Abbreviations

CI: confidence interval
CR: complete response
DCR: disease control rate
ECOG: Eastern Cooperative Oncology Group
GSEA: gene set enrichment analysis
HR: hazard ratio
LDH: lactic dehydrogenase
NSCLC: non-small-cell lung cancer
NSE: neuron-specific enolase
ORR: objective response rate
OS: overall survival
PFS: progression-free survival
PR: partial response
RECIST 1.1: Response Evaluation Criteria in Solid Tumors version 1.1
SCC: squamous cell carcinoma
SCLC: small cell lung cancer
SD: stable disease
TCGA: The Cancer Genome Atlas
TKI: tyrosine kinase inhibitor
